# KRAS Mutation Subtypes, Co-Mutations, PD-L1 Expression, and Survival Outcomes in Non-Small Cell Lung Cancer

**DOI:** 10.3390/jcm15135236

**Published:** 2026-07-04

**Authors:** Nesrin Gürçay, Funda Demirağ, Müzeyyen Burcu Kaplan Yılmaz, İlknur Öz, Tuba İnal Cengiz, Abdulkadir Koçanoğlu, Serdar Karakaya, Ömer Faruk Demir

**Affiliations:** 1 Department of Pathology, Ankara Atatürk Sanatoryum Training and Research Hospital, Ankara 06280, Turkey; fundademirag@yahoo.com.tr (F.D.); dr.mburcukaplan@gmail.com (M.B.K.Y.); nurilkoz95@gmail.com (İ.Ö.); 2 Department of Chest Diseases, Ankara Atatürk Sanatoryum Training and Research Hospital, Ankara 06280, Turkey; tubainalcengiz@gmail.com; 3 Department of Medical Oncology, Ankara Atatürk Sanatoryum Training and Research Hospital, Ankara 06280, Turkey; kadirkocanoglu@hotmail.com (A.K.); drserdarkarakaya@gmail.com (S.K.); 4 Department of Thoracic Surgery, Ankara Atatürk Sanatoryum Training and Research Hospital, Ankara 06280, Turkey; ofdemir@erciyes.edu.tr

**Keywords:** KRAS, NSCLC, PD-L1, immunotherapy, co-mutation, STK11, TP53

## Abstract

**Background:** KRAS mutations are among the most common oncogenic drivers in non-small cell lung cancer (NSCLC) and are associated with substantial molecular and immunological heterogeneity. However, the clinicopathological associations and prognostic relevance of KRAS mutation subtypes and co-occurring genomic alterations in relation to PD-L1 expression and survival outcomes remain incompletely understood, particularly in the immunotherapy era. **Methods:** This retrospective single-center study included 93 KRAS-mutant NSCLC patients identified among 543 consecutively sequenced cases between March 2024 and March 2025. KRAS mutation subtypes, co-mutations involving TP53, STK11, and KEAP1, PD-L1 expression status, clinicopathological features, and survival outcomes were evaluated. Overall survival was assessed using Kaplan–Meier analysis and Cox proportional hazards regression models. **Results:** KRAS mutations were detected in 17.1% of NSCLC patients. G12C was the most frequent KRAS subtype (38.7%), followed by G12V (18.3%) and G12D (14.0%). Co-occurring mutations were identified in 73.1% of cases, most commonly involving TP53 (40.9%) and STK11 (33.3%). PD-L1 expression was negative in 48.4% of patients, low in 28.0%, and high in 23.7%. No significant association was identified between KRAS mutation subtype and PD-L1 expression (*p* = 0.663). STK11-mutated tumors demonstrated a trend toward lower PD-L1 expression levels compared with STK11 wild-type tumors. However, none of the molecular variables retained independent prognostic significance. Immunotherapy was associated with significantly prolonged overall survival (median OS: 24 vs. 7 months, *p* = 0.013) and remained independently associated with improved survival in multivariate analysis (HR: 0.376, 95% CI: 0.204–0.694, *p* = 0.002). Advanced-stage disease independently predicted worse survival outcomes (HR: 13.43, 95% CI: 1.81–99.79, *p* = 0.011). **Conclusions:** KRAS mutation subtypes and co-occurring genomic alterations demonstrated limited independent prognostic significance in this real-world NSCLC cohort. In contrast, immunotherapy was associated with improved overall survival in this retrospective cohort. These findings should be interpreted as observational and hypothesis-generating rather than evidence of predictive treatment benefit. Larger prospective studies integrating genomic and immune biomarkers are warranted.

## 1. Introduction

Non-small cell lung cancer (NSCLC) remains the leading cause of cancer-related mortality worldwide and accounts for the majority of lung cancer diagnoses and deaths [1]. In recent years, the management of NSCLC has shifted toward precision oncology, with treatment strategies increasingly guided by molecular alterations and immune biomarkers [2]. Advances in molecular profiling technologies and targeted therapies have substantially improved the therapeutic landscape of thoracic oncology.

Among oncogenic drivers, KRAS mutations represent the most frequent molecular alteration in NSCLC, particularly in smoking-associated lung adenocarcinomas, with reported frequencies ranging from 25% to 30% [3,4]. KRAS mutations result in constitutive activation of downstream signaling pathways, including MAPK and PI3K–AKT, thereby promoting tumor proliferation, survival, and resistance to apoptosis [5]. However, KRAS-mutant NSCLC is increasingly recognized as a biologically heterogeneous disease rather than a single molecular entity. Distinct KRAS mutation subtypes, including G12C, G12V, and G12D, have been associated with differences in signaling pathway activation, tumor biology, and therapeutic responsiveness [6,7,8,9].

The recent development of KRAS G12C inhibitors, including sotorasib and adagrasib, has further increased the clinical relevance of KRAS molecular characterization in NSCLC [10,11]. Despite these advances, the prognostic implications of KRAS subtype heterogeneity remain incompletely understood, particularly in the era of immune checkpoint inhibition.

Programmed death-ligand 1 (PD-L1) expression has become a major predictive biomarker in NSCLC and plays a central role in guiding immunotherapy selection [12]. Several studies have demonstrated that KRAS-mutant tumors may exhibit increased PD-L1 expression, higher tumor mutational burden, and enhanced immunogenicity, suggesting potential biological interactions with the tumor immune microenvironment [13,14]. Nevertheless, the relationship between KRAS mutation subtypes and PD-L1 expression remains controversial, with conflicting findings reported across studies [15,16].

Another important source of heterogeneity in KRAS-mutant NSCLC is the presence of co-occurring genomic alterations, particularly involving TP53, STK11, and KEAP1. Previous studies have suggested that these co-mutations may influence tumor immune microenvironment characteristics and therapeutic response. KRAS–TP53 co-mutated tumors have been associated with increased inflammatory activity and enhanced responsiveness to immunotherapy, whereas KRAS–STK11 and KRAS–KEAP1 alterations have been linked to immune resistance and poorer clinical outcomes [17,18,19,20]. Although several studies have investigated the clinical implications of KRAS molecular heterogeneity, the relative contribution of KRAS mutation subtypes, co-mutations, and PD-L1 expression to survival outcomes remains uncertain [21]. In addition, real-world datasets integrating molecular profiling, immunotherapy exposure, and survival analyses remain relatively limited.

Despite these previous studies, relatively few real-world cohorts have simultaneously evaluated KRAS mutation subtypes, common co-mutations, PD-L1 expression, treatment exposure, and survival outcomes within a single molecularly characterized NSCLC population. Therefore, the present study was designed to address this gap by providing integrated real-world data from a routine clinical practice setting.

Therefore, the aim of the present study was to investigate the relationships between KRAS mutation subtypes, common co-mutations (TP53, STK11, and KEAP1), PD-L1 expression, and clinical outcomes in a real-world, molecularly characterized single-center cohort of NSCLC patients. Rather than establishing definitive prognostic or predictive biomarkers, our objective was to provide exploratory real-world evidence regarding these molecular and clinicopathological associations in the immunotherapy era.

This study was designed to complement the existing literature by providing real-world clinical data rather than establishing definitive prognostic or predictive conclusions.

## 2. Materials and Methods

### 2.1. Study Design and Patient Selection

This retrospective single-center study included patients diagnosed with non-small cell lung cancer (NSCLC) who underwent next-generation sequencing (NGS) analysis at Ankara Atatürk Sanatoryum Training and Research Hospital between March 2024 and March 2025. Among 543 consecutively analyzed NSCLC cases, 93 patients harboring KRAS mutations were identified and included in the final study cohort.

Clinical and pathological data, including age, sex, smoking history, histological subtype, disease stage, treatment modalities, progression status, and survival outcomes, were retrospectively retrieved from electronic medical records. Treatment decisions were made by the treating medical oncologists according to contemporary institutional practice and national reimbursement policies. Patients with PD-L1 tumor proportion scores (TPS) < 50% generally received platinum-based chemotherapy combined with immunotherapy as first-line treatment, whereas patients with PD-L1 TPS ≥ 50% were typically treated with pembrolizumab or atezolizumab monotherapy. KRAS G12C-targeted therapy (sotorasib) was available only for a very limited number of patients because it was not routinely reimbursed during the study period and therefore was not expected to materially influence the overall survival analyses. Histopathological classification was performed according to the current World Health Organization (WHO) classification of thoracic tumors, and tumor staging was based on the TNM staging system (8th edition) [22].

Overall survival (OS) was defined as the interval between the date of diagnosis and the date of death or last follow-up. Patients alive at the last follow-up were censored at the date of the most recent clinical evaluation.

The study was approved by the Scientific Research Ethics Committee of Ankara Atatürk Sanatoryum Training and Research Hospital (Approval No: 2024-BÇEK/440; Approval Date: 24 December 2025).

### 2.2. Histopathological Evaluation and PD-L1 Assessment

All hematoxylin–eosin-stained slides were reviewed by experienced thoracic pathologists. Histological subtypes were determined whenever sufficient tissue was available; however, a substantial proportion of cases consisted of small biopsy specimens, limiting comprehensive histological subtyping.

PD-L1 expression was evaluated by immunohistochemistry using validated clinical assays (22C3 pharmDx and SP263). Both assays are routinely used in clinical practice and are considered analytically comparable for NSCLC PD-L1 assessment. Tumor proportion score (TPS) was used for interpretation, and PD-L1 expression was categorized as follows:

- Negative: <1%
- Low expression: 1–49%
- High expression: ≥50% [23]

PD-L1 staining was independently evaluated by two experienced thoracic pathologists according to manufacturer-recommended scoring criteria.

### 2.3. Next-Generation Sequencing Analysis

Tumor-rich areas containing adequate viable tumor tissue were selected for molecular analysis. Samples were considered adequate for molecular testing if they contained at least 20% tumor cellularity and an estimated minimum of 200 viable tumor cells, following pathological review and tumor area selection. DNA and RNA were extracted from formalin-fixed paraffin-embedded (FFPE) tumor samples. Nucleic acid quantity and quality were assessed using the Qubit 4 fluorometer (Thermo Fisher Scientific, Waltham, MA, USA) to determine suitability for sequencing analysis.

Targeted next-generation sequencing was performed using the QIAGEN GeneReader platform (QIAGEN, Hilden, Germany) or the Illumina MiSeq/MiniSeq system (Illumina, San Diego, CA, USA) according to routine clinical laboratory practice. Targeted sequencing was carried out using QIAGEN GeneReader panel PCR kits and/or QIAGEN AISeq Targeted DNA/RNA panels.

The targeted DNA panel included the following genes: ALK, AKT1, AKT2, AKT3, ATM, ARID1A, ATRX, BRAF, CDKN2A, CTNNB1, DDR2, EGFR, ERBB2, ERBB4, ESR1, FAT1, FGFR1, FGFR2, FGFR3, FLT3, HRAS, KEAP1, KIT, KRAS, MAP2K1, MET, NF1, NOTCH1, NOTCH2, NOTCH4, NRAS, PDGFRA, PIK3CA, PTEN, PTPRD, PTPRT, RB1, RET, ROS1, RICTOR, SETD2, STK11, TP53, SMARCA2, and SMARCB1.

Bioinformatic analysis and variant interpretation were performed using QIAGEN Clinical Insight Analyze, QIAGEN Clinical Insight Analyze Universal, and QIAGEN Clinical Insight Interpret interfaces. Detected variants were comparatively evaluated using multiple genomic databases, including HGMD, COSMIC, 1000 Genomes Frequency Database, and Ingenuity Knowledge Base.

Variants with a variant allele frequency (VAF) below 5% or sequencing depth below 300× were excluded from analysis. Variant classification and clinical interpretation were performed according to AMP/ASCO/CAP 2017 guidelines [24]. Routine clinical laboratory quality-control procedures were applied throughout nucleic acid extraction, library preparation, sequencing, and bioinformatic analysis in accordance with the manufacturer’s recommendations. Targeted sequencing was performed using a validated workflow designed to achieve a minimum sequencing depth of approximately 1000×. According to our laboratory quality assurance protocol, the target performance criterion is for more than 90% of target regions to achieve a minimum coverage of 300× before clinical interpretation.

Co-occurring mutations involving TP53, STK11, and KEAP1 were specifically evaluated because they represent the most common and clinically well-established co-mutations in KRAS-mutant NSCLC. Although all genes included in the targeted NGS panel were analyzed, molecular alterations other than TP53, STK11, and KEAP1 occurred at low frequencies in the KRAS-mutant cohort. Therefore, separate statistical analyses of these less frequent co-mutations were not considered appropriate because of insufficient subgroup sizes.

### 2.4. Statistical Analysis

Statistical analyses were performed using IBM SPSS Statistics version 27.0 (IBM Corp., Armonk, NY, USA).

Categorical variables were compared using Pearson’s chi-square test or Fisher’s exact test, as appropriate. Overall survival was estimated using the Kaplan–Meier method, and survival differences between groups were assessed using the log-rank test.

Univariate and multivariate Cox proportional hazards regression analyses were performed to identify independent prognostic factors associated with survival outcomes. Variables with *p* < 0.10 in univariate analysis were included in the multivariate model. Candidate variables initially evaluated in the univariable Cox regression analyses included age, sex, smoking status, histological subtype, disease stage, PD-L1 expression, KRAS mutation subtype, common co-mutations (TP53, STK11, and KEAP1), and immunotherapy exposure. Variables with *p* < 0.10 in the univariable analyses were entered into the multivariable Cox proportional hazards model using the Enter method.

Multivariate logistic regression analysis was additionally performed to evaluate factors associated with disease progression. Results were reported as hazard ratios (HRs) or odds ratios (ORs) with corresponding 95% confidence intervals (CIs). Disease progression was analyzed as a binary outcome because precise dates of radiologic progression were not consistently available in this retrospective cohort, precluding reliable time-to-event analyses such as progression-free survival.

A two-sided *p*-value < 0.05 was considered statistically significant.

## 3. Results

### 3.1. Patient Characteristics

A total of 543 patients with NSCLC underwent next-generation sequencing (NGS) analysis during the study period. Among these, 93 patients (17.1%) harboring KRAS mutations were identified and included in the study cohort.

The mean age of the KRAS-mutant cohort was 66.1 ± 8.0 years (range: 36–87 years). The majority of patients were male (*n* = 82, 88.2%) and had adenocarcinoma histology (*n* = 87, 93.5%), whereas 6 patients (6.5%) were diagnosed with squamous cell carcinoma.

Most patients presented with advanced-stage disease (stage III–IV, *n* = 75, 80.6%). Regarding smoking status, 68.8% of patients were heavy smokers, 12.9% were light smokers, and 18.3% were never-smokers.

During follow-up, 46 patients (49.5%) experienced disease progression and 47 patients (50.5%) died. The median follow-up duration was 8 months (range: 0–52 months; mean: 11.4 ± 11.1 months).

In terms of treatment, 72 patients (77.4%) received chemotherapy, while 40 patients (43.0%) underwent immunotherapy. Baseline clinicopathological characteristics are summarized in Table 1.

### 3.2. Molecular and Pathological Characteristics

KRAS mutation subtypes were distributed as follows: G12C in 36 patients (38.7%), G12V in 17 patients (18.3%), G12D in 13 patients (14.0%), and other KRAS subtypes in 27 patients (29.0%).

Co-occurring genomic alterations were identified in 68 patients (73.1%). TP53 was the most frequently co-mutated gene (40.9%), followed by STK11 (33.3%) and KEAP1 (6.5%).

PD-L1 expression in the KRAS-mutant cohort was negative in 45 patients (48.4%), low (1–49%) in 26 patients (28.0%), and high (≥50%) in 22 patients (23.7%).

Among adenocarcinoma cases, a substantial proportion of tumors were represented by small biopsy specimens, limiting detailed histological subtyping in some patients.

The molecular and pathological characteristics of the KRAS-mutant cohort are summarized in Table 2.

### 3.3. Association Between KRAS Mutation Subtypes, Co-Mutations, and PD-L1 Expression

No statistically significant association was observed between KRAS mutation subtypes and PD-L1 expression status (Pearson χ^2^, *p* = 0.663). Similarly, subgroup analyses evaluating individual KRAS subtypes, including G12C, G12D, and G12V, did not demonstrate significant differences in PD-L1 expression profiles (all *p* > 0.05). The distribution of PD-L1 expression according to KRAS mutation subtype is presented in Table 2.

Co-mutation status was also not significantly associated with PD-L1 expression. TP53-mutated tumors tended to demonstrate higher rates of elevated PD-L1 expression compared with TP53 wild-type tumors; however, this association did not reach statistical significance (*p* = 0.249).

Interestingly, STK11-mutated tumors demonstrated a trend toward lower PD-L1 expression levels and reduced frequency of high PD-L1 expression compared with STK11 wild-type tumors (9.7% vs. 30.6%, respectively), although the overall association did not reach conventional statistical significance (Pearson χ^2^, *p* = 0.065).

No significant association was identified between KEAP1 alterations and PD-L1 expression (*p* = 0.259).

### 3.4. Survival Analysis

**Figure 1:**
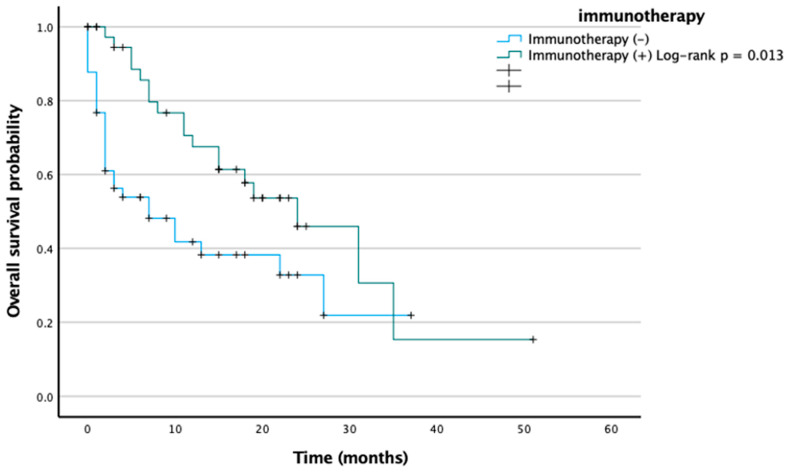
Kaplan–Meier curves for overall survival according to immunotherapy status. Patients treated with immunotherapy demonstrated significantly prolonged overall survival compared with patients who did not receive immunotherapy (median OS: 24 vs. 7 months; log-rank *p* = 0.013). Abbreviations: OS, overall survival. +: censored observations.

Kaplan–Meier survival analysis demonstrated significantly improved overall survival in patients receiving immunotherapy compared with those who did not receive immunotherapy (median OS: 24 vs. 7 months, log-rank *p* = 0.013) (Figure 1).

No statistically significant differences in overall survival were observed according to KRAS mutation subtype classification (*p* = 0.660). Likewise, TP53 and STK11 mutation status were not independently associated with significant survival differences (*p* = 0.330 and *p* = 0.284, respectively), although mutation-positive subgroups tended to exhibit shorter survival durations.

### 3.5. Cox Proportional Hazards Regression Analysis

In the univariable Cox regression analysis, age, disease stage, smoking status, and immunotherapy exposure were associated with overall survival (Table 3). Variables with *p* < 0.10 in the univariable analyses were entered into the multivariable Cox proportional hazards model.

In the multivariable analysis, advanced-stage disease remained independently associated with worse overall survival (HR: 13.642, 95% CI: 1.842–101.023, *p* = 0.011). Immunotherapy remained independently associated with improved overall survival (HR: 0.504, 95% CI: 0.272–0.935, *p* = 0.030). Increasing age (HR: 1.043, 95% CI: 1.001–1.087, *p* = 0.047) and smoking status (HR: 0.640, 95% CI: 0.457–0.898, *p* = 0.010) were also independently associated with overall survival.

The results of Cox proportional hazards regression analyses are summarized in Table 3.

Variables with *p* < 0.10 in the univariable Cox proportional hazards analysis were entered into the multivariable model using the Enter method—indicates variables not included in the multivariable model.

Reference categories: female sex, stage I–II, never smoker, TP53/STK11/KEAP1 wild-type, and no immunotherapy.

### 3.6. Logistic Regression Analysis for Disease Progression

Multivariate logistic regression analysis revealed that immunotherapy was independently associated with a significantly reduced risk of disease progression (OR: 0.114, 95% CI: 0.039–0.332, *p* < 0.001).

Additionally, early-stage disease was associated with a lower risk of progression compared with advanced-stage disease (OR: 0.137, 95% CI: 0.024–0.770, *p* = 0.024).

Smoking status was not significantly associated with disease progression (*p* = 0.715).

The results of logistic regression analysis are presented in Table 4.

## 4. Discussion

In the present study, we comprehensively evaluated the associations between KRAS mutation subtypes, co-occurring genomic alterations, PD-L1 expression, and clinical outcomes in a real-world cohort of NSCLC patients. Within the limitations of this cohort, no statistically significant associations were observed between KRAS mutation subtypes and PD-L1 expression or survival outcomes. These findings should be interpreted as exploratory and do not exclude the possibility of clinically relevant associations that may become apparent in larger adequately powered cohorts. Our findings should therefore be interpreted as complementary to the existing body of real-world evidence rather than as definitive evidence establishing the prognostic significance of KRAS molecular heterogeneity. By integrating KRAS mutation subtypes, co-mutation profiles, PD-L1 expression, treatment exposure, and survival outcomes within a single molecularly characterized real-world cohort, our study provides additional evidence from routine clinical practice while underscoring the need for validation in larger multicenter studies.

KRAS-mutant NSCLC is increasingly recognized as a biologically heterogeneous disease comprising multiple molecular subtypes with distinct biological behaviors. Previous studies have suggested that specific KRAS variants, particularly G12C and G12V, may exhibit differential signaling pathway activation, tumor microenvironment characteristics, and therapeutic responsiveness [6,7,8,9,25]. However, despite these biological differences, we did not observe significant survival differences among KRAS subtypes. These findings are generally consistent with previous real-world studies that also did not identify clear prognostic differences among KRAS mutation subtypes [21,26].

The recent development of KRAS G12C inhibitors has substantially increased the clinical relevance of KRAS molecular characterization in NSCLC. Clinical trials evaluating sotorasib and adagrasib have demonstrated promising activity in KRAS G12C-mutant tumors and established KRAS as a therapeutically actionable target [10,11]. Nevertheless, the prognostic implications of KRAS subtype heterogeneity in the immunotherapy era remain controversial. Our findings indicate that, within this cohort, KRAS mutation subtype was not independently associated with overall survival despite recognized biological heterogeneity.

Another important finding of our study was the absence of a significant association between KRAS mutation subtype and PD-L1 expression. Previous reports investigating this relationship have yielded conflicting results. Some studies suggested that KRAS-mutated tumors, particularly KRAS G12C tumors and those harboring concurrent TP53 alterations, may exhibit enhanced immunogenicity and improved responsiveness to immune checkpoint inhibitors [13,16], whereas others failed to identify subtype-specific differences [15,17]. Our findings are more consistent with studies reporting no significant association between KRAS subtype and PD-L1 expression.

Notably, STK11-mutated tumors in our cohort demonstrated a trend toward lower PD-L1 expression and reduced frequency of high PD-L1 expression compared with STK11 wild-type tumors, although this association did not reach conventional statistical significance. This observation is in agreement with previous reports suggesting that STK11 alterations may be associated with an immunologically “cold” tumor microenvironment and reduced responsiveness to immune checkpoint inhibitors. However, these biological mechanisms were not directly evaluated in the present study and therefore should be interpreted as literature-based hypotheses rather than findings derived from our cohort [17,19,20]. Similarly, TP53-mutated tumors tended to exhibit higher PD-L1 expression levels, which is also concordant with previously reported associations between TP53 alterations and increased tumor immunogenicity [18]. Previous studies have suggested that TP53 alterations may be associated with increased tumor immunogenicity; however, these mechanistic observations were not specifically investigated in the present study. Accordingly, these biological mechanisms should be regarded as contextual information from the literature rather than observations derived from the present study.

Co-occurring genomic alterations represent a major source of molecular heterogeneity in KRAS-mutant NSCLC. TP53, STK11, and KEAP1 alterations have all been implicated in modulating tumor immune biology and therapeutic response. Although co-mutations were common in our cohort, they were not independently associated with survival outcomes. This may partly reflect the relatively limited sample size and subgroup heterogeneity, particularly for KEAP1-mutated tumors.

One of the clinically relevant findings of this study was the observed association between immunotherapy exposure and improved overall survival. However, because of the retrospective observational design and the lack of randomized treatment allocation, this finding should be interpreted as an association rather than evidence of a causal treatment effect [13,14].

Importantly, our findings suggest that the treatment strategy may have a greater impact on clinical outcomes than KRAS molecular heterogeneity within this retrospective cohort. However, this observation should be interpreted cautiously because of the observational design and the potential influence of residual confounding.

Consistent with established evidence, advanced-stage disease remained independently associated with worse survival outcomes, emphasizing the continued prognostic importance of disease stage despite advances in molecular oncology and systemic therapy.

One important strength of the present study is its reflection of real-world thoracic oncology practice. Our cohort included predominantly advanced-stage patients and a substantial number of small biopsy specimens, which reflects routine clinical practice and enhances the real-world applicability of our findings.

## 5. Limitations

Several limitations should be acknowledged. First, the retrospective single-center design may introduce inherent selection bias. Second, the relatively limited cohort size, particularly within individual KRAS mutation subgroups and co-mutation categories, may have limited the statistical power to detect modest prognostic associations. Therefore, the absence of statistically significant differences should not be interpreted as evidence of a true lack of biological or clinical relevance. Although our findings provide real-world data from a well-characterized single-center cohort, they should be considered exploratory and hypothesis-generating. Validation in larger multicenter cohorts and independent external sequencing datasets is warranted to further clarify the prognostic significance of KRAS molecular heterogeneity in NSCLC.

Furthermore, because detailed information regarding the exact timing of immunotherapy initiation, treatment sequencing, ECOG performance status, and several other potential confounding variables was not consistently available, advanced approaches such as time-dependent Cox regression, landmark analyses, or propensity score adjustment could not be performed. Therefore, the observed association between immunotherapy and overall survival should be interpreted cautiously and considered hypothesis-generating.

As a retrospective single-center study, formal sensitivity analyses and a STROBE-style participant flow diagram were not available.

The relatively short median follow-up duration may have limited the maturity and reliability of the overall survival analyses. Therefore, the survival findings should be interpreted with appropriate caution and require confirmation with longer follow-up in future studies. PD-L1 expression was assessed using two clinically validated assays (22C3 pharmDx and SP263). Although these assays are generally considered analytically comparable, potential inter-assay variability cannot be completely excluded.

## 6. Conclusions

In addition, because disease progression was analyzed as a binary endpoint using logistic regression, which should be interpreted in light of this methodological limitation. Therefore, only overall survival was analyzed, and the findings regarding immunotherapy should be considered exploratory and hypothesis-generating. Future prospective studies incorporating these clinically relevant endpoints are warranted to validate our findings.

## Figures and Tables

**Table 1:** Baseline Clinicopathological Characteristics of the KRAS-Mutant Cohort (*n* = 93).

Variable	Value
Age, years	66.1 ± 8.0 (range: 36–87)
Male, *n* (%)	82 (88.2%)
Female, *n* (%)	11 (11.8%)
Adenocarcinoma, *n* (%)	87 (93.5%)
Squamous cell carcinoma, *n* (%)	6 (6.5%)
Stage I–II, *n* (%)	18 (19.4%)
Stage III–IV, *n* (%)	75 (80.6%)
Never-smoker, *n* (%)	17 (18.3%)
Light smoker (<20 pack-years), *n* (%)	12 (12.9%)
Heavy smoker (≥20 pack-years), *n* (%)	64 (68.8%)
Chemotherapy, *n* (%)	72 (77.4%)
Immunotherapy, *n* (%)	40 (43.0%)
Disease progression, *n* (%)	46 (49.5%)
Death, *n* (%)	47 (50.5%)
Median follow-up, months	8 (range: 0–52)
Mean follow-up, months	11.4 ± 11.1

Abbreviations: SD, standard deviation.

**Table 2:** (**A**). Molecular and Pathological Characteristics of the KRAS-Mutant NSCLC Cohort (*n* = 93). (**B**). Association Between KRAS Mutation Subtypes and PD-L1 Expression.

**A**					
**Variable**			***n* (%)**		
G12C			36 (38.7%)		
G12V			17 (18.3%)		
G12D			13 (14.0%)		
Other KRAS subtypes			27 (29.0%)		
Any co-mutation			68 (73.1%)		
TP53			38 (40.9%)		
STK11			31 (33.3%)		
KEAP1			6 (6.5%)		
Negative PD-L1 (<1%)			45 (48.4%)		
Low PD-L1 (1–49%)			26 (28.0%)		
High PD-L1 (≥50%)			22 (23.7%)		
**B**					
**KRAS Subtype**	**Negative** (**<1%**)	**Low** (**1–49%**)	**High** (**≥50%**)	**Total**	***p*-Value**
G12C	16 (44.4%)	11 (30.6%)	9 (25.0%)	36	
G12V	11 (64.7%)	2 (11.8%)	4 (23.5%)	17	
G12D	7 (53.8%)	3 (23.1%)	3 (23.1%)	13	
Other KRAS subtypes	11 (40.7%)	10 (37.0%)	6 (22.2%)	27	
Total	45 (48.4%)	26 (28.0%)	22 (23.7%)	93	0.663

Abbreviations: PD-L1, programmed death-ligand 1. Statistical analysis: Pearson chi-square test. Fisher–Freeman–Halton exact test confirmed the absence of significant association (*p* = 0.678).

**Table 3:** Univariable and multivariable Cox proportional hazards analyses for overall survival.

Variable	Univariable HR (95% CI)	*p* Value	Multivariable HR (95% CI)	*p* Value
Age	1.042 (1.002–1.084)	0.038	1.043 (1.001–1.087)	0.047
Sex	0.985 (0.439–2.209)	0.971	—	—
Histology	0.236 (0.032–1.721)	0.154	—	—
Stage (III–IV vs. I–II)	12.510 (1.721–90.929)	0.013	13.642 (1.842–101.023)	0.011
Smoking status	0.567 (0.405–0.793)	<0.001	0.640 (0.457–0.898)	0.010
PD-L1 expression	1.137 (0.808–1.600)	0.462	—	—
KRAS mutation subtype	0.874 (0.684–1.115)	0.278	—	—
TP53 co-mutation	1.092 (0.611–1.952)	0.767	—	—
STK11 co-mutation	1.588 (0.885–2.851)	0.121	—	—
KEAP1 co-mutation	1.549 (0.552–4.350)	0.406	—	—
Immunotherapy	0.610 (0.339–1.096)	0.098	0.504 (0.272–0.935)	0.030

**Table 4:** Multivariate Logistic Regression Analysis for Disease Progression.

Variable	OR	95% CI	*p*-Value
Immunotherapy	0.114	0.039–0.332	<0.001
Early stage disease (I–II vs. III–IV)	0.137	0.024–0.770	0.024
Smoking status	0.894	0.492–1.624	0.715

Abbreviations: OR, odds ratio; CI, confidence interval.

## Data Availability

The datasets generated and/or analyzed during the current study are available from the corresponding author on reasonable request.
